# MCM4 expression is associated with high-grade histology, tumor progression and poor prognosis in urothelial carcinoma

**DOI:** 10.1186/s13000-023-01392-y

**Published:** 2023-09-22

**Authors:** Go Kobayashi, Tetsutaro Hayashi, Kazuhiro Sentani, Naohiro Uraoka, Takafumi Fukui, Aya Kido, Narutaka Katsuya, Akira Ishikawa, Takashi Babasaki, Yohei Sekino, Hiroyuki Nose, Koji Arihiro, Nobuyuki Hinata, Naohide Oue

**Affiliations:** 1 Department of Pathology, Kure-Kyosai Hospital, Federation of National Public Service Personnel Mutual Aid Associations, Hiroshima, Japan; 2 Department of Molecular Pathology, Graduate School of Biomedical and Health Sciences, Hiroshima University, -2-3 Kasumi, Minami-ku, Hiroshima, 734-8551 Japan; 3 Department of Urology, Graduate School of Biomedical and Health Sciences, Hiroshima University, Hiroshima, Japan; 4 Department of Urology, Kure-Kyosai Hospital, Federation of National Public Service Personnel Mutual Aid Associations, Hiroshima, Japan; 5 Department of Anatomical Pathology, Hiroshima University Hospital, Hiroshima, Japan

**Keywords:** MCM4, Urothelial carcinoma, Clinicopathological significance, Immunohistochemistry, Urine cytology, Immunocytochemistry

## Abstract

**Background:**

We previously reported Minichromosome maintenance 4 (MCM4) overexpression in gastric cancer. However, the clinicopathological significance of MCM4 in urothelial carcinoma (UC) has not been investigated. To clarify the clinicopathological significance of MCM4 in UC, we investigated MCM4 expression with immunohistochemistry (IHC).

**Methods:**

We analyzed the expression and distribution of MCM4 in 124 upper tract urothelial carcinoma (UTUC) samples by IHC. Additionally, using 108 urine samples, we analyzed MCM4 Immunocytochemistry (ICC) expression in urine cytology.

**Results:**

In normal urothelium, MCM4 expression was weak or absent. Meanwhile, the strong nuclear expression of MCM4 was observed in UTUC tissues, and it was detected in 77 (62%) of a total of 124 UTUC cases. MCM4-positive UTUC cases were associated with nodular/flat morphology, high grade, high T stage, and poor prognosis. Moreover, MCM4 expression was significantly higher in the invasive front than in the tumor surface. Similar results were also obtained in TCGA bladder cancer cohort. Additionally, MCM4 expression was associated with high expression of Ki-67, HER2, EGFR, and p53 in UTUC. Among representative cancer-related molecules, MCM4 had an independent predictive value for progression-free survival and high-grade UC. ICC for MCM4 was also performed on urine cytology slides and showed that the nuclear expression of MCM4 was more frequently found in UC cells than in non-neoplastic cells. The diagnostic accuracy of urine cytology was improved by combining MCM4 immunostaining with cytology.

**Conclusion:**

These results suggest that MCM4 might be a useful predictive biomarker for high-grade histology, tumor progression and poor prognosis in UC. Moreover, ICC for MCM4 might be helpful for UC detection as additional markers in the cytomorphology-based diagnosis.

**Supplementary Information:**

The online version contains supplementary material available at 10.1186/s13000-023-01392-y.

## Background

Urothelial carcinoma (UC) is a common type of malignant disease and occurs in the upper urinary tract (renal pelvis and ureter) or lower urinary tract (bladder and urethra). The prognosis of advanced UC remains poor: 5-year cancer-specific survival (CSS) in patients with pT4 disease is < 10% in both bladder cancer (BC) and upper tract urothelial carcinoma (UTUC) [1–3]. Despite this, little is known about biomarkers for the prognosis and diagnosis of UC, especially UTUC. Recently, a number of immune markers (e.g., HER2, EGFR, CD44v9, PD-L1, Nectin-4, MUC1, claspin) have been reported as predictive markers for poor prognosis or high-grade UC in patients with UTUC [4–8]. Prognostic biomarkers are important for guiding therapeutic options and surveillance strategies [5]. Although various clinicopathological parameters, including increasing pathological stage and high tumor grade have been demonstrated as poor prognostic factors, their predictive accuracy remains limited due to individual variation [1, 2, 9]. Thus, it is necessary to identify new prognostic markers and potential therapeutic targets in UC.

Currently, as the correct grading and staging of UC are quite important, biopsy for histopathological examination or urine cytology has an essential role in daily practice [10]. Because performing biopsy in UTUC cases is invasive and difficult in comparison to BC, urine cytology plays a key role in the diagnosis of UTUC [10–12]. However, its diagnostic accuracy remains controversial [12]. Thus, the identification of a novel immunomarker to improve the diagnostic accuracy of urine cytology would be highly desirable.

We previously identified the minichromosome maintenance 4 (MCM4) gene from spheroid forming gastric cancer (GC) cells and reported its important role in GC progression [13]. MCM4 belongs to the minichromosome maintenance protein family, and regulates DNA replication and genome stability by unwinding double strands of DNA and facilitating replication fork progression [14–16]. MCM4 has recently been reported as a new prognostic biomarker in some cancers, including GC, breast cancer and hepatocellular carcinoma [13, 17, 18]. However, MCM4 expression in UC has not yet been investigated, and to our knowledge it is still largely unknown whether MCM4 expression can be applied to cytology.

In the present study, we investigated the clinicopathological significance of MCM4 in UC for the first time using surgical UTUC tissue specimens. The reason why we used UTUC cohort is that analyzing UTUC samples has an advantage in comparing correlation between molecule expression and clinicopathological factors in several stages of UTUC treated by RNU without the effect of the modifications such as TUR. We also evaluated the association between MCM4 expression and representative cancer-related molecules. Furthermore, using patient urine samples, we analyzed MCM4 expression in urine cytology.

## Methods

### Tissue samples

All samples were obtained with patient consent, and the present study was approved by the Ethical Committee for Human Genome Research of Hiroshima University (authorization number: E-589) and Kure-Kyosai Hospital (authorization number: 31–47). To investigate the clinicopathological significance of MCM4 in UC, medical records of patients who underwent radical nephroureterectomy (RNU) for UTUC at Hiroshima University Hospital between April 1999 and May 2019 were retrospectively reviewed. Patients with neo-adjuvant chemotherapy were excluded from this study, leaving 124 patients for inclusion. The clinicopathological data was obtained from medical records. According to the 8th edition of the American Joint Committee on Cancer/Union for International Cancer Control (AJCC/UICC) TNM classification (2017), pathology specimens were examined and re-reviewed for staging. To evaluate the tumor grade, we used the 2004 WHO/ISUP 2-tier grading system.

The mean and standard deviation of the patient’s age distribution were 71.4 and 9.8, with males accounting for 76% (94 patients) of the cohort. During the follow-up period, disease progression was observed in 30 (24%) patients, 18 (15%) of whom died of the disease. The endpoints of this study were CSS and progression-free survival (PFS). Disease progression was defined as any documented first relapse in the lymph nodes or evidence of distant metastasis, whereas bladder cancer recurrence or contralateral upper tract UC were not considered as disease progression. Our follow-up protocol consisted of a urine analysis and chest-abdomen-pelvis CT with or without contrast every 3–6 months for at least five years, according to the preferences of each physician. The last follow-up date was August 1, 2020.

### Urine cytology

A total of 108 cytology samples were collected from patients at Kure-Kyosai Hospital (Hiroshima, Japan) between 2020 and 2022. The type of urine samples was voided urines (*n* = 85), bladder washings (*n* = 2) and ureter/renal pelvic washings (*n* = 21), which were clinicopathologically confirmed as benign urothelium (*n* = 57), bladder UC (*n* = 32) and UTUC (*n* = 19). BC and UTUC were histologically divided into low-grade UC (*n* = 10), high-grade UC (*n* = 24), variants of UC (*n* = 3) and unknown histological type (*n* = 14). The cytological diagnosis was made according to the 2015 Japan Reporting System for Urinary Cytology, and it was divided into the following 4 categories: negative for malignancy (Negative), atypical urothelial cells (Atypical), suspicious for malignancy (Suspicious) and malignant (Malignant). Although the detection of high grade UC is given priority in Japan Reporting System, it can also be used for the detection of low grade UC and other tumors. Thus, it was classified as malignant and diagnosed as LGUC if the large number of cells that three-dimensional structures or papillary fragments with fibrovascular cores had nuclei of the same size. If there was a possibility of LGUC, it was often classified as atypical or suspicious. The distribution of the cytological diagnoses was as follows: Negative (*n* = 39), Atypical (*n* = 38), Suspicious (*n* = 13) and Malignant (*n* = 18). The 2 categories of Suspicious and Malignancy were considered cytology-positive.

Urine cytology was performed using BD SurePath™ (BD, Becton Dickinson, Ltd., Wokingham, UK) liquid-based cytology (LBC). To analyze immunocytochemistry (ICC) for MCM4, the residual LBC samples fixed with CytoRich Red solution (BD, Becton Dickinson, Ltd., Franklin Lakes, NJ, USA) were reserved for several weeks after the cytological diagnosis, and we selected samples containing a sufficient number of cells which were subsequently confirmed as benign or malignant.

### Immunohistochemistry (IHC)

Immunohistochemical staining was performed on 1 or 2 representative tumor blocks using a Bond-3 automated immunostainer platform (Leica Biosystems, Bannockburn, IL) according to the manufacturer’s protocol. Antigen retrieval was performed using heat-based antigen retrieval EDTA-based high pH epitope retrieval buffer (pH 9, Leica ER2 buffer), with a heating time of 20 min, with the temperature of the equipment set to 99 °C. Peroxidase activity was blocked for 5 min with a Bond Polymer Refine kit. Sections were incubated with a mouse polyclonal anti-MCM4 antibody (dilution 1:100) for 30 min. Stain detection was performed using a bond polymer detection kit (Refine, Leica) for 8 min. The sections were incubated with DAB for 10 min for color reaction and then counterstained with 0.1% haematoxylin from a bond polymer detection kit. Negative controls were created by omission of the primary antibody.

MCM4 expression was scored as positive or negative. MCM4 expression was evaluated at a 10% threshold in concordance with our previous IHC analysis of GC [13]. Using this definition, two observers (G. K. and K. S.) without knowledge of the clinicopathological parameters or patient outcomes independently reviewed the immunoreactivity of each specimen. The evaluation of Ki-67 programmed death-ligand 1 (PD-L1), CD44 variant 9 (CD44v9), human epidermal growth factor receptor 2 (HER2), epidermal growth factor receptor (EGFR), fibroblast growth factor receptor 3 (FGFR3), p53, GATA binding protein 3 (GATA3) and cytokeratin 5/6 (CK5/6) was described previously [7, 8, 19].

Immunocytochemistry (ICC) was performed on urine cytology slides using BD SurePath™ with a Bond-III automated immunostainer platform using the above-described immunohistochemistry protocol. We evaluated the percentage of MCM4 staining in urothelial cells, regardless of the presence or absence of cytologic atypia, and counted at least 100 cells per slide.

### TCGA databases analysis

The RNA-Seq expression of MCM4 genes and clinico-pathological data of bladder urothelial carcinoma (BLCA) were downloaded from http://xena.ucsc.edu/cite-us. To investigate the relationship between MCM4 and prognosis in BC, we downloaded the public dataset from OncoLnc (https://peerj.com/articles/cs-67/). The correlation between MCM4 and MKI67, EGFR, TP53 and FGFR3 gene expression in BLCA was exprored with TIMER2.0 (http://timer.cistrome.org/).

### Statistical analysis

All statistical analyses were performed using the SPSS software program (SPSS Inc., Chicago, IL, USA). Correlations between clinicopathological parameters and the expression of MCM4 were analyzed using Fisher’s exact test. Kaplan-Meier survival curves were constructed for MCM4-positive and MCM4-negative patients, and the survival rates of the two groups were compared. Differences between survival curves were tested for statistical significance by a log-rank test. Univariate and multivariate Cox proportional hazards regression analyses were performed to evaluate the associations between clinical covariates or various representative molecules and survival. Univariate and multivariate logistic regression analyses were performed to identify the independent predictive marker for high-grade UC. The comparison of MCM4 expression between two groups was evaluated by the Wilcoxon signed-rank test and Mann-Whitney U test. The diagnostic accuracy, including the sensitivity, specificity, positive and negative predictive values, receiver operating characteristic (ROC) curve and area under the ROC curve (AUC) were calculated. P values of < 0.05 were considered to indicate statistical significance. An ROC curve analysis was also used to determine the optimal cut-off value using Youden’s index for the expression of MCM4 in urine cytology that was correlated with the final diagnosis.

## Results

### Expression of MCM4 in UTUC and its relationship with clinicopathological parameters

**Fig. 1:**
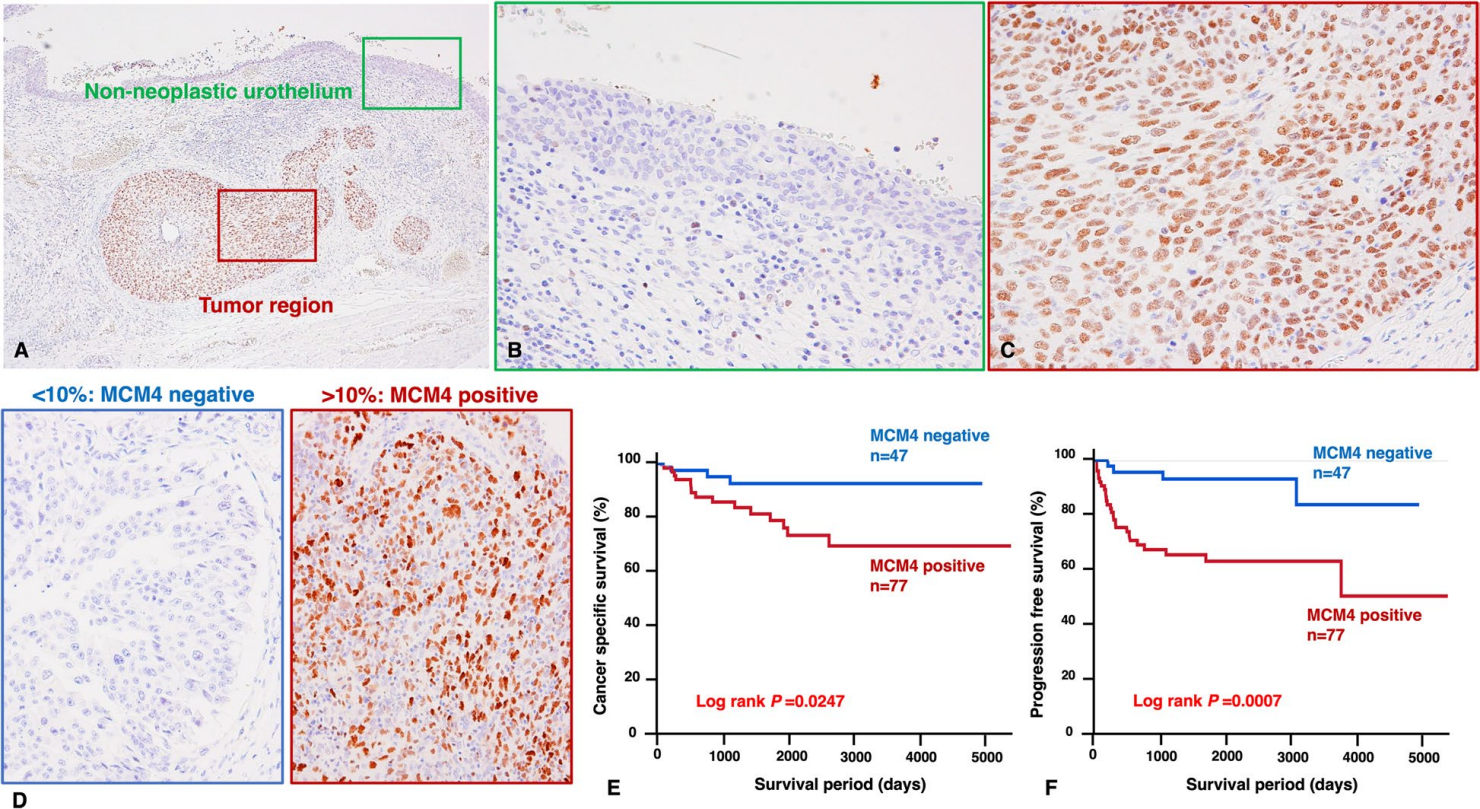
Immunohistochemical analysis of MCM4 in upper tract urothelial carcinoma (UTUC). **A** Representative staining images of MCM4 in urothelial carcinoma and adjacent non-neoplastic urothelium tissues (original magnification ×400). **B** High-magnification image of MCM4 expression in non-neoplastic urothelium. **C** High-magnification image of MCM4 expression in tumor region. **D** Representative staining images of MCM4-positive staining and ANXA10-negative staining. Kaplan-Meier plot of CSS (**E**) and PFS (**F**) of patients with UTUC according to tumor MCM4 expression. (Immunohistochemistry, original magnification ×40 (**A**), ×200 (**B, C** and **D**))

We first performed immunohistochemical staining of 124 UTUC tissue samples. In non-neoplastic urothelium, the staining of MCM4 was weak or absent (Fig. 1A, B). The strong nuclear expression of MCM4 was detected in UTUC tissues (Fig. 1A, C). When > 10% of tumor cells were stained, immunostaining was considered positive for MCM4 (Fig. 1D). The expression of MCM4 was detected in 77 (62%) of the total of 124 UTUC cases. Next, we analyzed the relationship between MCM4 expression and various clinicopathological characteristics. MCM4-positive UTUC cases were associated with ages > 70 years (*P* = 0.0410), ureter UC (*P* = 0.0309), nodular/flat morphology (*P* = 0.0086), high tumor grade (*P* < 0.0001) and high pathological T stage (*P* < 0.0001) (Table 1).

**Table 1:** Relationship between MCM4 expression and clinicopathological parameters in 124 upper tract urothelial carcinoma cases obtained by immunohistochemistry

	MCM4 expression		*P* value
	High (%)	Low	
**Age**			
≤ 70 (*n* = 56)	29 (52%)	27	**0.0410**
> 70 (*n* = 68)	48 (62%)	20	
Sex			
Female (*n* = 30)	22 (73%)	8	0.1951
Man (*n* = 94)	55 (59%)	39	
**Location**			
Renal pelvis (*n* = 63)	32 (51%)	31	**0.0309**
Ureter (*n* = 57)	42 (74%)	15	
Both (*n* = 4)	3 (75%)	1	
Lateralization			
Right (*n* = 59)	37 (63%)	22	> 0.9999
Left (*n* = 65)	40 (62%)	35	
**Morphology**			
Papillary (*n* = 65)	36 (51%)	34	**0.0086**
Nodular/Flat (*n* = 54)	41 (76%)	13	
Histological classification			
Pure UC (*n* = 97)	61 (63%)	36	0.3219
Variants (*n* = 11)	9 (82%)	2	
**Histological grade**			
Low grade (*n* = 55)	21 (38%)	34	**< 0.0001**
High grade (*n* = 69)	56 (81%)	13	
Pathological T stage			
pTa/is/1 (*n* = 61)	24 (39%)	37	**< 0.0001**
pT2/3/4 (*n* = 63)	53 (84%)	10	

*P* values were calculated with Fisher’s exact test

Bold values show the statistical significance at the *P* < 0.05 level

### Relationship between MCM4 expression and the prognosis of UC

We next performed a Kaplan–Meier survival analysis. MCM4 expression was significantly associated with decreased CSS (*P =* 0.0247, log-rank test; Fig. 1E) and PFS (*P =* 0.0007, log-rank test; Fig. 1F). We also performed univariate and multivariate Cox proportional hazards analyses. In the univariate analysis, age, morphology, histological grade, T grade and MCM4 expression were associated with poor survival. Although MCM4 was not an independent prognostic predictor for CSS, MCM4 was marginally significantly associated with decreased PFS (*P* = 0.0813) in the multivariate model (Table S1).

**Fig. 2:**
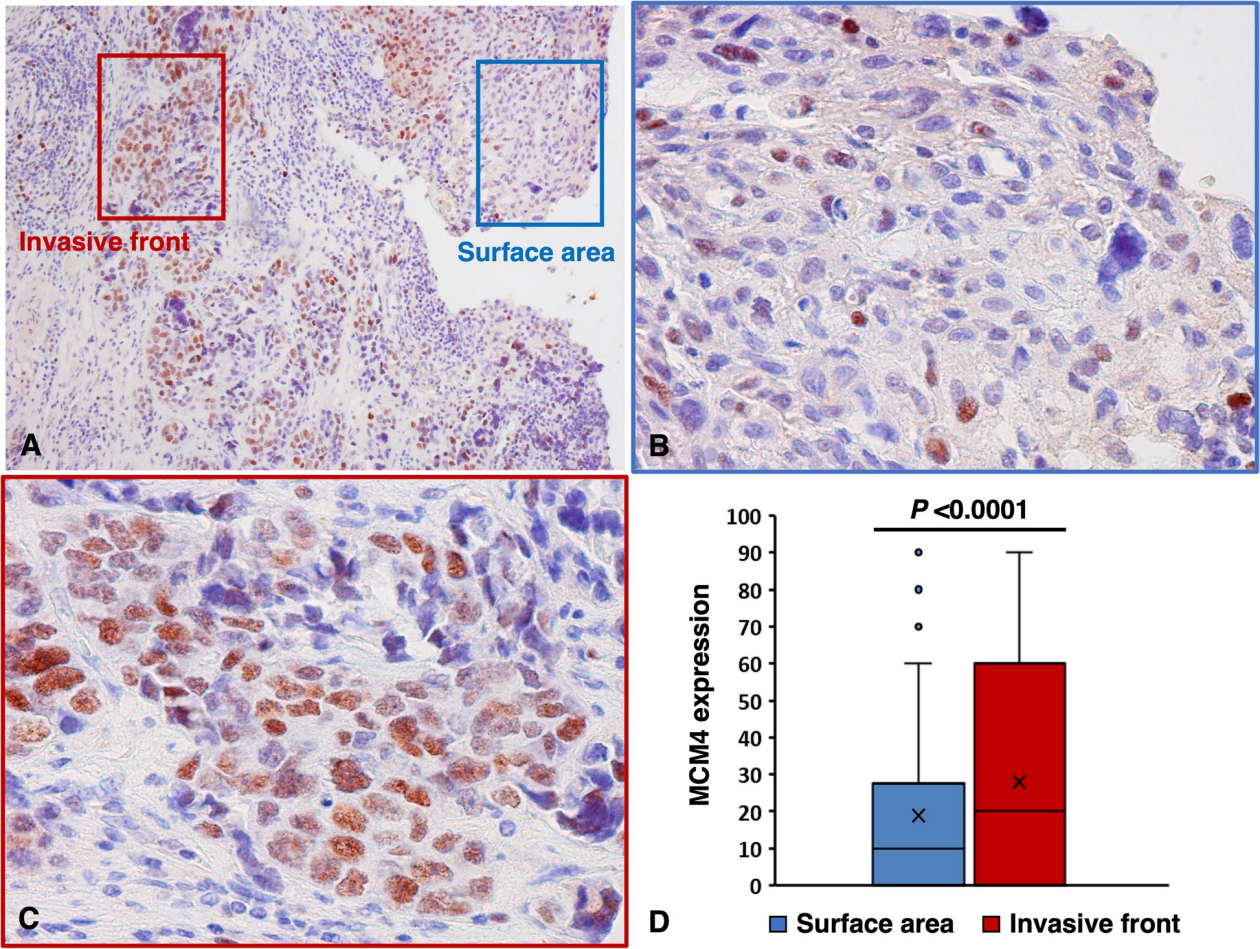
Analysis of MCM4 expression in the surface area and invasive front. **A** Low-power magnification of MCM4 in UC tissues including the surface area and invasive front. **B** MCM4 expression on tumor surface. **C** MCM4 expression in tumor invasive front. **D** Comparison of MCM4 expression between the surface area and invasive front in all upper tract UC tissues (*n* = 124). Statistical significance was determined by the Wilcoxon signed-rank test. (Immunohistochemistry, original magnification ×100 (**A**), ×400 (**B** and **C**))

Moreover, MCM4 expressed cells were frequently observed in the deeper invasive region compared to the surface of the tumor (Fig. 2A-C). We also examined MCM4 expression in the surface area and deeply invasive front. The expression of MCM4 was significantly higher in the invasive front than on the surface (*P* < 0.0001, Wilcoxon signed-rank test; Fig. 2D). There was no significant different between these locations in the pTa/is/1, and overexpression of MCM4 was significantly observed in invasive front in pT2/3/4 tumors (Supplementary Fig. 1).

### Correlation between MCM4 expression and various cancer-related molecules

**Fig. 3:**
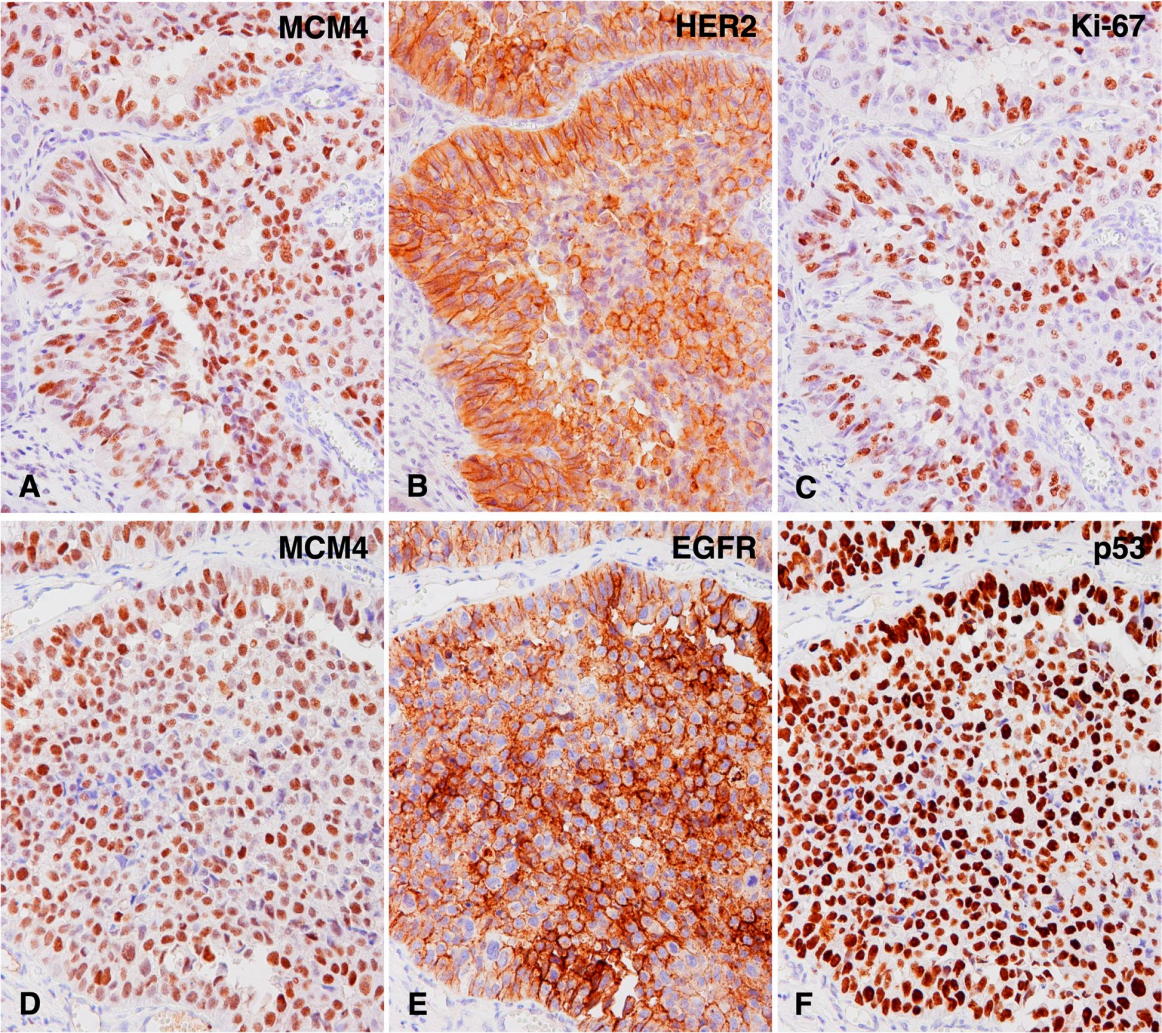
Immunohistochemical analysis of MCM4 and various molecules in consecutive tumor sections of upper tract urothelial carcinoma. **A** The nuclear expression of MCM4. **B** The membranous expression of HER2. **C** The nuclear expression of Ki-67. **D** The nuclear expression of MCM4. **E** The membranous expression of HER2. **F** The nuclear expression of p53. (Immunohistochemistry, original magnification ×200 (**A**, **B**, **C**, **D**, **E** and **F**)

Although we showed that MCM4 could contribute to tumor progression in UTUC, the molecules with which MCM4 is associated remain unclear. We therefore investigated the relationship between MCM4 expression and various cancer-related molecules, including Ki-67, PD-L1 CD44v9, HER2, EGFR, FGFR3, p53, GATA3 and CK5/6 in 124 UTUC cases. We revealed that MCM4 expression was significantly associated with the high expression of Ki-67 (*P* = 0.0036), HER2 (*P* = 0.0355), EGFR (*P* = 0.0140) and p53 (*P* = 0.0011) (Fig. 3; Table 2).

**Table 2:** Relationship between MCM4 expression and various cancer related molecules in 124 upper tract urothelial carcinoma cases

	MUC1 expression		*P* value
	High (%)	Low	
**Ki-67**			
Positive (20%<)	29 (67%)	6	**0.0036**
Negative (20%>)	48 (54%)	41	
PD-L1 in TCs			
Positive	14 (78%)	4	0.1904
Negative	63 (59%)	43	
PD-L1 in TILs			
Positive	26 (68%)	12	0.4228
Negative	51 (59%)	35	
CD44v9			
Positive	16 (67%)	8	0.8129
Negative	50 (63%)	29	
**HER2**			
Positive	18 (82%)	4	**0.0355**
Negative	59 (58%)	43	
**EGFR**			
Positive	25 (81%)	6	**0.0181**
Negative	52 (55%)	41	
FGFR3			
Positive	25 (69%)	11	0.3139
Negative	52 (59%)	36	
**p53**			
Positive	31 (89%)	4	**< 0.0001**
Negative	46 (52%)	43	
GATA3			
Positive	68 (61%)	43	0.7647
Negative	9 (69%)	4	
CK 5/6			
Positive	17 (65%)	9	0.8213
Negative	60 (61%)	38	

*Abbreviations*: *PD-L1 *Programmed death ligand 1, *TCs *Tumor cells, *TILs *Tumor-infiltrating lymphocyte, *HER2 *Human epidermal growth factor receptor type 2, *EGFR *Epidermal growth factor receptor, *FGFR3 *Fibroblast growth factor receptor 3

*P* values were calculated with Fisher’s exact test

Bold values show the statistical significance at the *P* < 0.05 level

### Comparison between MCM4 expression and various cancer‑related molecules for identifying an independent predictive marker of patient prognosis and high‑grade UC

We next investigated whether MCM4 has an independent predictive value for prognosis and high-grade UC compared to other markers. We performed univariate and multivariate Cox proportional hazards analyses of PFS and CSS. In the univariate analysis, MCM4, PD-L1 (TCs and TILs), CD44v9 and EGFR were associated with CSS, and MCM4, PD-L1 in TCs and CD44v9 were associated with PFS. In the multivariate models, there were no independent markers of CSS, while the expression of MCM4 (*P* = 0.0090) and CD44v9 (*P* = 0.0132) provided an independent prognostic marker for PFS (Table 3).

**Table 3:** Comparison of univariate and multivariate Cox proportional hazards analyses of progression-free survival and cancer-specific survival in MCM4 expression and other molecules

	Cancer specific survival				Progression free survival			
	Univariate analysis		Multivariate analysis		Univariate analysis		Multivariate analysis	
	HR (95% CI)	*P*	HR (95% CI)	*P*	HR (95% CI)	*P*	HR (95% CI)	*P*
MCM4								
Negative	1 (Reference)	**0.0366**	1 (Reference)	0.3838	1 (Reference)	**0.0022**	1 (Reference)	**0.0090**
Positive	3.76 (1.24–16.22)		1.80 (0.48–6.74)		5.19 (2.01–17.60)		5.07 (1.73–21.62)	
Ki-67								
Negative	1 (Reference)	0.0841			1 (Reference)	0.1166		
Positive	2.27 (0.87–5.76)				1.82 (0.83–3.78)			
PD-L1 in TCs								
Negative	1 (Reference)	**0.0012**	1 (Reference)	0.2213	1 (Reference)	**0.0093**	1 (Reference)	0.2009
Positive	4.82 (1.86–12.49)		2.10 (0.64–6.94)		2.97 (1.31–6.76)		1.85 (0.72–4.77)	
PD-L1 in TILs								
Negative	1 (Reference)	**0.0073**	1 (Reference)	0.1003	1 (Reference)	0.3858		
Positive	3.58 (1.41–9.10)		2.62 (0.83–8.26)		1.40 (0.65-3.00)			
CD44v9								
Negative	1 (Reference)	**0.0400**	1 (Reference)	0.1258	1 (Reference)	**0.0155**	1 (Reference)	**0.0132**
Positive	3.03 (1.00-8.73)		2.33 (0.79–6.86)		2.78 (1.17–6.27)		2.87 (1.20–6.51)	
HER2								
Negative	1(Reference)	0.8354			1(Reference)	0.6696		
Positive	1.14 (0.33–3.94)				1.25 (0.44–3.61)			
EGFR								
Negative	1(Reference)	0.3310			1(Reference)	0.1024		
Positive	1.63 (0.61–4.34)				1.89 (0.88–4.03)			
FGFR3								
Negative	1(Reference)	0.1954			1(Reference)	0.3400		
Positive	0.44 (0.13–1.52)				0.66 (0.28–1.54)			
p53								
Negative	1 (Reference)	0.3782			1 (Reference)	0.2433		
Positive	1.55 (0.54–4.01)				1.57 (0.70–3.29)			

Bold values show the statistical significance at the *P* < 0.05 level

*Abbreviations*: *HR *Hazard ratio, *CI *Confidence interval, *PD-L1 *Programmed death ligand 1, *TCs *Tumor cells, *TILs *Tumor-infiltrating lymphocyte, *HER2 *Human epidermal growth factor receptor type 2, *EGFR *Epidermal growth factor receptor, *FGFR3 *Fibroblast growth factor receptor 3

We next performed univariate and multivariate logistic regression analyses to identify an independent predictive marker of high-grade UC. In the univariate analysis, MCM4, Ki-67, PD-L1 (TCs and TILs), EGFR and p53 were associated with high grade UC, with MCM4 having the highest odds ratio. In the multivariate model, the expression of MCM4 (*P* = 0.0002) gave an independent predictive value for high-grade UC (Table 4 ).

**Table 4:** Comparison of univariate and multivariate logistic regression analyses for identification of independent predictive marker of high grade urothelial carcinoma in MCM4 expression and other molecules

	Prediction of high grade UC			
	Univariate analysis		Multivariate analysis	
	OR (95% CI)	*P*	OR (95% CI)	*P*
MCM4				
Negative	1 (Reference)	**< 0.0001**	1 (Reference)	**0.0002**
Positive	6.97 (3.09–15.71)		5.18 (2.11–12.70)	
Ki-67				
Negative	1 (Reference)	**0.0244**	1 (Reference)	0.8086
Positive	2.56 (1.10–5.94)		1.13 (0.41–3.11)	
PD-Ll in TCs				
Negative	1 (Reference)	**0.0073**	1 (Reference)	0.1311
Positive	4.81 (1.32–17.61)		2.98 (0.67–13.27)	
PD-L1 in TILs				
Negative	1(Reference)	**0.0200**	1 (Reference)	0.3668
Positive	2.57 (1.13–5.83)		1.60 (0.57–4.47)	
CD44v9				
Negative	1(Reference)	0.0768		
Positive	2.37 (0.88–6.34)			
HER2				
Negative	1(Reference)	0.0697		
Positive	2.46 (0.89–6.81)			
EGFR				
Negative	1 (Reference)	**0.0442**	1 (Reference)	0.5803
Positive	2.39 (1.01–5.74)		1.33 (0.48–3.64)	
FGFR3				
Negative	1 (Reference)	0.6996		
Positive	1.17 (0.53–2.56)			
p53				
Negative	1 (Reference)	**0.0004**	1 (Reference)	0.1382
Positive	4.68 (1.85–11.84)		2.19 (0.76–6.29)	

Bold values show the statistical significance at the *P* < 0.05 level.

*Abbreviations*: *OR O*dds ratio, *CI *Confidence interval, *PD-L1 *Programmed death ligand 1, *TCs *Tumor cells, *TILs *Tumor-infiltrating lymphocyte, *HER2 *Human epidermal growth factor receptor type 2, *EGFR *Epidermal growth factor receptor, *FGFR3 *Fibroblast growth factor receptor 3

### Comprehensive analysis of MCM4 expression in BC using the TCGA BLCA dataset

We then evaluated the relationship between MCM4 expression and several clinico-pathological parameters in the TCGA BLCA dataset (*n* = 453) using mRNA expression data. MCM4 expression was significantly present in BC tissue (*P <* 0.0001) (Supplementary Fig. 2A). In combination with clinicopathologic features, MCM4 expression was associated with high grade (*P <* 0.0001) and non-papillary morphology (*P* = 0.0059), while no statistical difference between MCM4 and stage in the TCGA BC data was found (Supplementary Fig. 2B-D). Other TCGA data sets by OncoLnc revealed that MCM4 expression was associated with poor survival (*P* = 0.0247, log-rank; Supplementary Fig. 2E). We continued checking MCM4 expression in the TCGA BC and other TCGA datasets with TIMER2.0, which revealed that MCM4 expression was associated with MKI67 (Ki-67) (*P <* 0.0001) and EGFR (*P <* 0.0001), marginally significantly associated with TP53 (*P* = 0.0510), and inversely associated with FGFR3 gene expression (*P <* 0.0001) (Supplementary Fig. 2G-J).

### Analysis of MCM4 expression in urine cytology samples

**Fig. 4:**
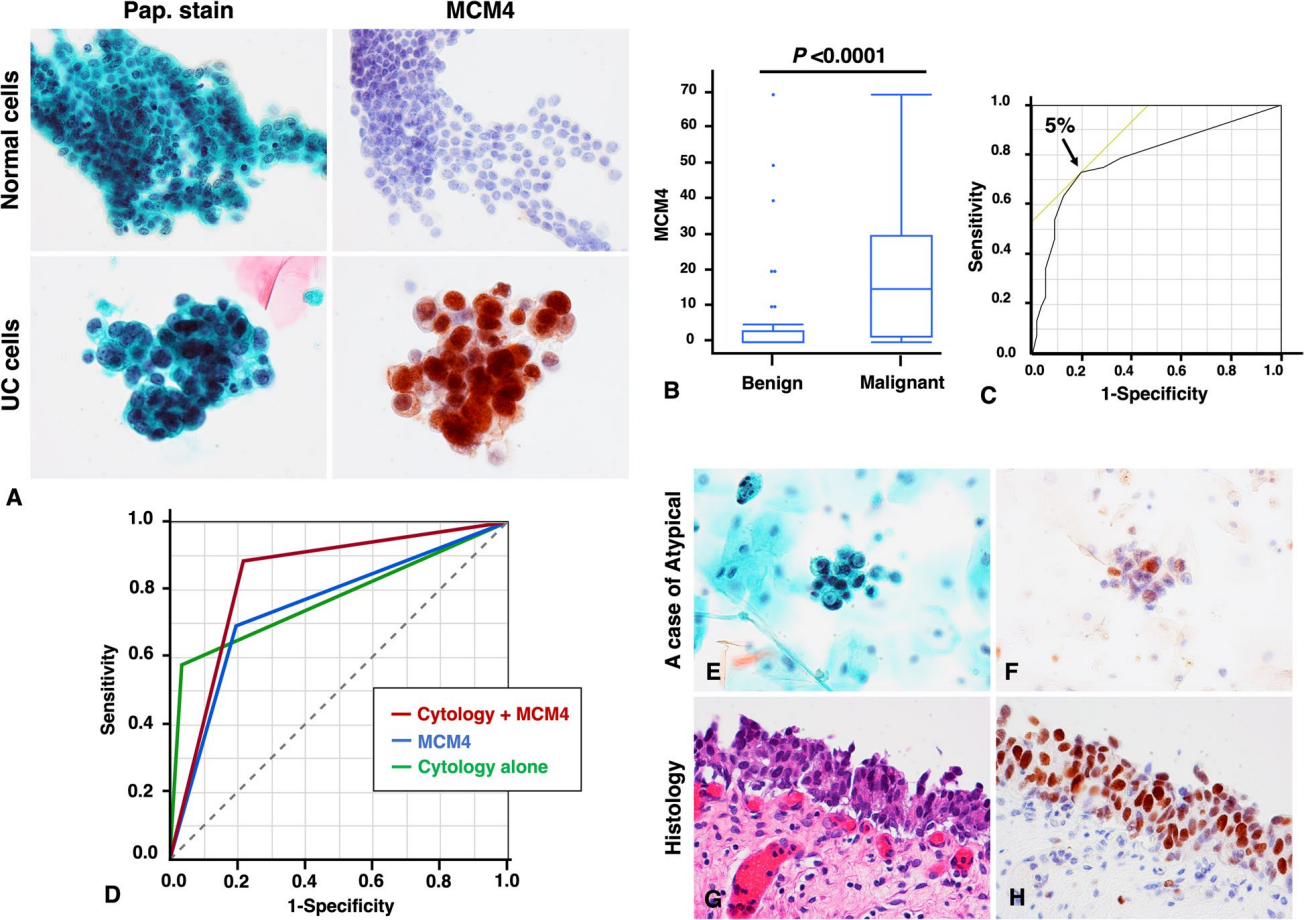
Analysis of the MCM4 expression in urine cytology. **A** Representative images of ICC for MCM4 in normal urothelial cells UC cells. **B** Comparison of MCM4 expressed-cells between benign and UC cases. Statistical significance was determined by the Mann-Whitney U test. **C** ROC analysis in ICC for MCM4 staining and diagnosis of UC. **D** ROC and area under the ROC curve (AUC) analysis of cytology, MCM4, cytology with MCM4 in the diagnosis of UC. **E** Papanicolaou stained slides and (**F**) ICC staining of MCM4 in a case with Atypical diagnosis, (**G**) histologically diagnosed as high grade UC (hematoxylin and eosin staining), and (**H**) tumor cells were found to be positive for MCM4 by immunohistochemistry. (Papanicolaou staining and ICC, original magnification ×400 (**A, E** and **F**), hematoxylin and eosin staining, original magnification ×400 (**G**), immunohistochemistry, original magnification ×400 (**H**)

Finally, we performed an immunocytochemical analysis of 108 urine cytology samples. The staining of MCM4 was weak or absent in non-neoplastic urothelial cells, whereas nuclear staining of MCM4 was found in atypical cells in the UC diagnosed case (Fig. 4A). We evaluated the percentage of MCM4-expressed cells and found that the MCM4-expressed cells were observed significantly more frequently in UC cases than in benign urothelium (*P <* 0.0001, Mann-Whitney U test; Fig. 4B).

We then conducted an ROC analysis to determine the cut-off value of the MCM4 immunocytostaining score to evaluate the diagnostic accuracy between cytology, MCM4 and the combination of cytology and MCM4. Since the optimal cut-off value was 5%, MCM4-positive was defined as strong staining of > 5% of urothelial cells for MCM4 (Fig. 4C). The test of the combination of cytology and MCM4 was considered to be positive if either or both cytology and MCM4 were positive, and negative if both cytology and MCM4 were negative. The results are shown in Table 5. The diagnostic accuracy of cytology combined with MCM4 was better compared to cytology alone. The ROC curve analysis showed that the AUC was 0.84 when cytology and MCM4 were combined (Fig. 4D).

**Table 5:** Diagnostic accuracy of MCM4 expression and cytology for the detection of urothelial carcinoma in urine cytology cases

	Cytology	MCM4	Cytology + MCM4
Sensitivity, % (95% CI)	58 (49–61)	69 (59–77)	89 (79–95)
Specificity, % (95% CI)	96 (89–99)	80 (71–88)	79 (70–84)
PPV, % (95% CI)	94 (80–99)	77 (65–86)	79 (71–85)
NPV, % (95% CI)	71 (65–73)	74 (65–81)	88 (78–95)
AUC (95% CI)	0.77 (0.69–0.83)	0.75 (0.66–0.82)	0.84 (0.75–0.89)

*Abbreviations*: *PPV *Positive predictive value, *NPV *Negative predictive value, *AUC *Area under the curve.

In the example case demonstrating the usefulness of MCM4, the cytological findings showed a few atypical cells with slightly enlarged nuclei, with the cytological classification being Atypical (Fig. 4E), while ICC showed that the cells were positive for MCM4 (Fig. 4F). Histological examination subsequently revealed the presence of high-grade UC in the bladder biopsy (Fig. 4G), and these tumor cells were also strongly immunohistochemically positive for MCM4 (Fig. 4H).

## Discussion

In the present study, our immunohistochemical analysis showed that MCM4 expression was detected in 77 (62%) of 124 UTUC cases and associated with nodular/flat morphology, high tumor grade and high pathological T grade. The overexpression of MCM4 was more frequently observed in the invasive front. MCM4 expression was significantly associated with decreased CSS and PFS. In addition, MCM4 was an independent prognostic marker for PFS compared to representative cancer-related markers. Taken together, these results suggest that MCM4 represents a useful predictor of tumor progression in UC.

UC is known to be a heterogeneous nature and often exhibit diversity in various pathological factors, including histological type, variants, the presence of carcinoma in situ and infiltrative patterns, which are closely associated with tumor progression and patient prognosis [20, 21]. In particular, the infiltrative pattern and tumor budding at the tumor invasion front are reported to be associated with high malignant potential and poor clinical outcome [21]. The present data revealed that MCM4 expression was significantly associated with poor prognosis and high in tumor invasive front. Therefore, MCM4 might contribute the development of characteristic histological structures in tumor invasive front, and further study will be needed on the relationship between MCM4, tumor heterogeneity and microenvironment in UC.

The present study demonstrated that MCM4 expression was associated with high Ki-67 labeling index in both UTUC and BC-TCGA data, which was consistent with previous studies showing MCM4 to be a proliferation marker in several cancers [22–24]. In fact, we previously demonstrated the functional role of MCM4 in significantly increasing cell proliferative ability in MCM4 siRNA-transfected gastric cancer [13]. In addition to Ki-67, the expression of MCM4 was positively correlated with p53. Mutations of the p53 gene and immunohistochemical positivity for p53 have been found in 40–60% of UCs and is associated with high cancer grade and stage [25, 26]. Generally, low-grade papillary UC predominantly follows the FGFR3 signaling pathway, while nodular/flat UC such as carcinoma in situ and high-grade invasive disease follows the TP53 pathway [27]. Our data showed that MCM4 expression was significantly associated with nodular/flat morphology and p53 expression. In addition, the TCGA data showed that MCM4 was negatively correlated with FGFR3 gene expression. Taken together, MCM4 could be a useful proliferation marker in UC and play a critical role in UC carcinogenesis by participating in the activation of TP53 pathway. Interestingly, MCM4-positive UTUC cases were associated with ureter UC (vs. renal pelvis). Previous studies have shown that ureter UC have worse survival than renal pelvis [28, 29]. Moreover, according to Fujii et al., UTUC can be divided into five DNA-based molecular subtypes such as hypermutated, TP53/MDM2, RAS, FGFR3, and triple negative, and 50.6% of ureter UC was TP53/MDM2 mutational subtype [30]. Since MCM4 expression was also associated with poor prognosis and p53 expression, there might be an association between MCM4 expression and the biology of ureter UC.

Several studies have shown that the expression of MCM4 in cancer is regulated by many factors [31, 32]. In the present study, we revealed that MCM4 expression was associated with HER2 and EGFR expression. HER2 and EGFR are also known as representative therapeutic targets in many malignancies, including UC [33, 34]. In UTUC, the overexpression of HER2 and EGFR are associated with a high tumor stage, high histological grade, and poor survival [5]. Interestingly, a recent study has demonstrated that a relationship between EGFR and MCM4 is observed in lung adenocarcinoma [35]. In addition, our previous study has confirmed the MCM4 was associated with AKT, ERK and EGFR pathways by functional analysis using GC cell lines [13]. Although further study is needed, these data indicate that MCM4 may be a promising predictor for HER2/EGFR targeting UC and may promote tumor progression by participating in the EGFR/HER2 signaling pathways in UC.

Clinically, as low grade UC has low malignant potential while high grade UC carries risk of disease progression, metastases and cause mortality, it is very important not to overlook high grade UC cases in particular [36–38]. Thus, there is a push to focus on diagnosing predominantly high grade UC in The Paris System [39, 40]. Previous studies have revealed that representative cancer-related molecules such as Ki-67, PD-L1, CD44v9, HER2, EGFR and p53 are associated with high grade UC [5, 26, 41–43]. As the present study revealed that MCM4 had an independent predictive value for high grade UC in comparison to these markers, MCM4 might be a potential biomarker for high grade UC in urine cytology. In general, the exfoliate cells observed as urine cytology samples are known to be derived from the surface area, while our present data showed that MCM4 expression levels in the surface was low especially in pT2/3/4 tumors, in comparison to the invasive front. However, the expression of MCM4 was often observed even in the surface area, and some expression levels of MCM4 were also present in the early stage of UTUC. Given the staining of MCM4 was weak or absent in the non-neoplastic urothelium, its expression levels in the surface were also considered to be high compared to normal tissues. In fact, our ICC analysis revealed that the MCM4-expressed cells were observed significantly more frequently in UC cases than in benign cases. Urine cytology has an important role in the evaluation of UC, with highly specificity but poor sensitivity [44]. In addition, the cytological classification of Atypical should be considered as neither a positive nor negative cytological result, and is one of the most frequently encountered problems in clinical practice [45, 46]. To address these problems, several ancillary tests such as UroVysion and uCyt +/immunoCyt have been developed [47, 48]. However, these tests often produce false positive results, and a limited number of facilities use them because they are associated with high cost and time consumption and require skilled technicians [49–51]. On the other hand, ICC can be performed quickly and easily in everyday practice and has the advantage of being related to the morphology of cells in urine cytology [47, 52]. In the present study, although the sensitivity of cytology alone for the detection of UC—including UTUC—was relatively low, its diagnostic accuracy was improved by the combined use of ICC for MCM4 and cytology. Interestingly, immunostaining of p53 and Ki-67 has been reported as a useful marker for the diagnosis of UC based on urine cytology, and we have shown that MCM4 was correlated with both Ki-67 and p53 [53]. These findings indicate that MCM4 expression has possible application as an ancillary marker in the cytomorphology-based diagnosis.

The present study included some limitations. First, this study was a retrospective study, and a prospective series will be necessary to confirm the present data. Second, the detailed molecular mechanism involved in the relationships between MCM4 expression, HER2, EGFR and p53 in UTUC are still unclear. Therefore, further extensive study will be required to elucidate the molecular activity in tumor cell biology. Third, although we have demonstrated possible diagnostic applications of MCM4 ICC, the number of included patients was limited. Thus, a further study will be undertaken to extensively investigate the test performance in order to improve the diagnostic accuracy of urine cytology in the near future. Another possible limitation is that a few false-positives of MCM4 ICC with benign cases were present in this study, so the application of MCM4 would be ideal for use as an adjunct to cytomorphology-based diagnosis.

## Conclusions

We investigated MCM4 expression in UTUC and revealed that MCM4 might be a useful predictive marker for high grade, tumor progression and poor prognosis. In addition, ICC for MCM4 might be helpful for UC detection in urine cytology.

### Supplementary Information

**Additional file 1: Supplementary Table 1. **Univariate and multivariate Cox proportional hazards analyses of progression free survival and cancer-specific survival in 124 upper tract urothelial carcinoma cases.

**Additional file 2: Supplementary Fig. 1. **Comparison of MCM4 expression between the surface area and invasive front in pTa/is/1 (n = 61) and pT2/3/4 (n = 63) tumors respectively.

**Additional file 3: Supplementary Fig. 2. **MCM4 expression in bladder cancer (BC) using TCGA dataset. (A) MCM4 expression in normal and tumor tissues. (B, C, D) MCM4 expression associated with clinicopathological features. Statistical significance was determined by the Mann-Whitney U test. (E) Kaplan-Meier analysis of BC patients with high and low MCM4 expression. (G, H, I, J) In silico analysis of the correlation between MCM4, MKI67, EGFR, TP53 and FGFR3 gene expression. Statistical significance was determined by the Spearman rank correlation test.

## Data Availability

All research data and material will be made available upon request. Most data are included in the main manuscript.

## Abbreviations

AUC: Area under the receiver operating characteristic curve
BC: Bladder cancer
CSS: Cancer-specific survival
GC: Gastric cancer
ICC: Immunocytochemistry
IHC: Immunohistochemistry
LBC: Liquid-based cytology
MCM4: Minichromosome maintenance 4
PFS: progression-free survival
ROC: Receiver operating characteristic curve and area under the ROC curve (AUC)
UC: Urothelial carcinoma
UTUC: Upper tract urothelial carcinoma
