# Supplementary material for: MCM4 expression is associated with high-grade histology, tumor progression and poor prognosis in urothelial carcinoma
Source: Diagn Pathol. 2023 Sep 22;18:106. doi: 10.1186/s13000-023-01392-y (PMC10515259; doi:10.1186/s13000-023-01392-y)
Supplement: Supplementary file 1 — Additional file 1: Supplementary Table 1. Univariate and multivariate Cox proportional hazards analyses of progression free survival and cancer-specific survival in 124 upper tract urothelial carcinoma cases. [file 13000_2023_1392_MOESM1_ESM.docx]

| **Supplementary Table 1. Univariate and multivariate Cox proportional hazards analyses of progression free survival and cancer-specific survival in 124 upper tract urothelial carcinoma cases.** | | | | | | | | |
| --- | --- | --- | --- | --- | --- | --- | --- | --- |
|  | Cancer specific survival | | | | Progression free survival | | | |
|  | Univariate analysis | | Multivariate analysis | | Univariate analysis | | Multivariate analysis | |
|  | HR (95% CI) | *P* | HR (95% CI) | *P* | HR (95% CI) | *P* | HR (95% CI) | *P* |
| Age |  |  |  |  |  |  |  |  |
| ≤ 70 | 1 (Reference) | **0.0175** | 1 (Reference) | **0.0219** | 1 (Reference) | 0.3007 |  |  |
| > 70 | 3.87 (1.38-13.72) |  | 3.78 (1.33-13.73) |  | 1.48 (0.71-3.17) |  |  |  |
| Sex |  |  |  |  |  |  |  |  |
| Female | 1 (Reference) | 0.2919 |  |  | 1 (Reference) | 0.3102 |  |  |
| Male | 0.58 (0.22-1.70) |  |  |  | 0.67 (0.31-1.53) |  |  |  |
| Morphology |  |  |  |  |  |  |  |  |
| Papillary | 1 (Reference) | **0.0016** | **1 (Reference)** | **0.1486** | 1 (Reference) | **<.0001** | 1 (Reference) | **0.0212** |
| Nodular/Flat | 6.01 (2.15-21.17) |  | 2.46 (0.79-9.67) |  | 5.03 (2.31-29.10) |  | 2.97 (1.23-7.96) |  |
| Grade |  |  |  |  |  |  |  |  |
| Low grade | 1(Reference) | **0.0221** | 1 (Reference) | 0.9305 | 1(Reference) | **0.0029** | 1 (Reference) | 0.8887 |
| High grade | 3.67 (1.31-12.95) |  | 1.07 (0.26-5.20) |  | 3.63 (1.63-9.17) |  | 1.08 (0.40-3.19) |  |
| T grade |  |  |  |  |  |  |  |  |
| pTa/is/1 | 1(Reference) | **0.0031** | 1 (Reference) | 0.0150 | 1(Reference) | **0.0002** | 1 (Reference) | 0.1468 |
| pT2/3/4 | 20.94 (4.30-377.20) |  | 14.66 (2.43-284.28) |  | 5.38 (2.34-14.56) |  | 2.22 (0.79-6.99) |  |
| MCM4 |  |  |  |  |  |  |  |  |
| Negative | 1 (Reference) | **0.0366** | 1 (Reference) | 0.8870 | 1 (Reference) | **0.0022** | 1 (Reference) | 0.0813 |
| Positive | 3.76 (1.24-16.22) |  | 0.89 (0.20-4.97) |  | 5.19 (2.01-17.60) |  | 2.79 (0.96-10.18) |  |
| Bold values show the statistical significance at the *P* < 0.05 level.  Abbreviations: HR, hazard ratio; CI, confidence interval. | | | | | | | | |
