# Supplementary figures and images for: MCM4 expression is associated with high-grade histology, tumor progression and poor prognosis in urothelial carcinoma
Source: Diagn Pathol. 2023 Sep 22;18:106. doi: 10.1186/s13000-023-01392-y (PMC10515259; doi:10.1186/s13000-023-01392-y)

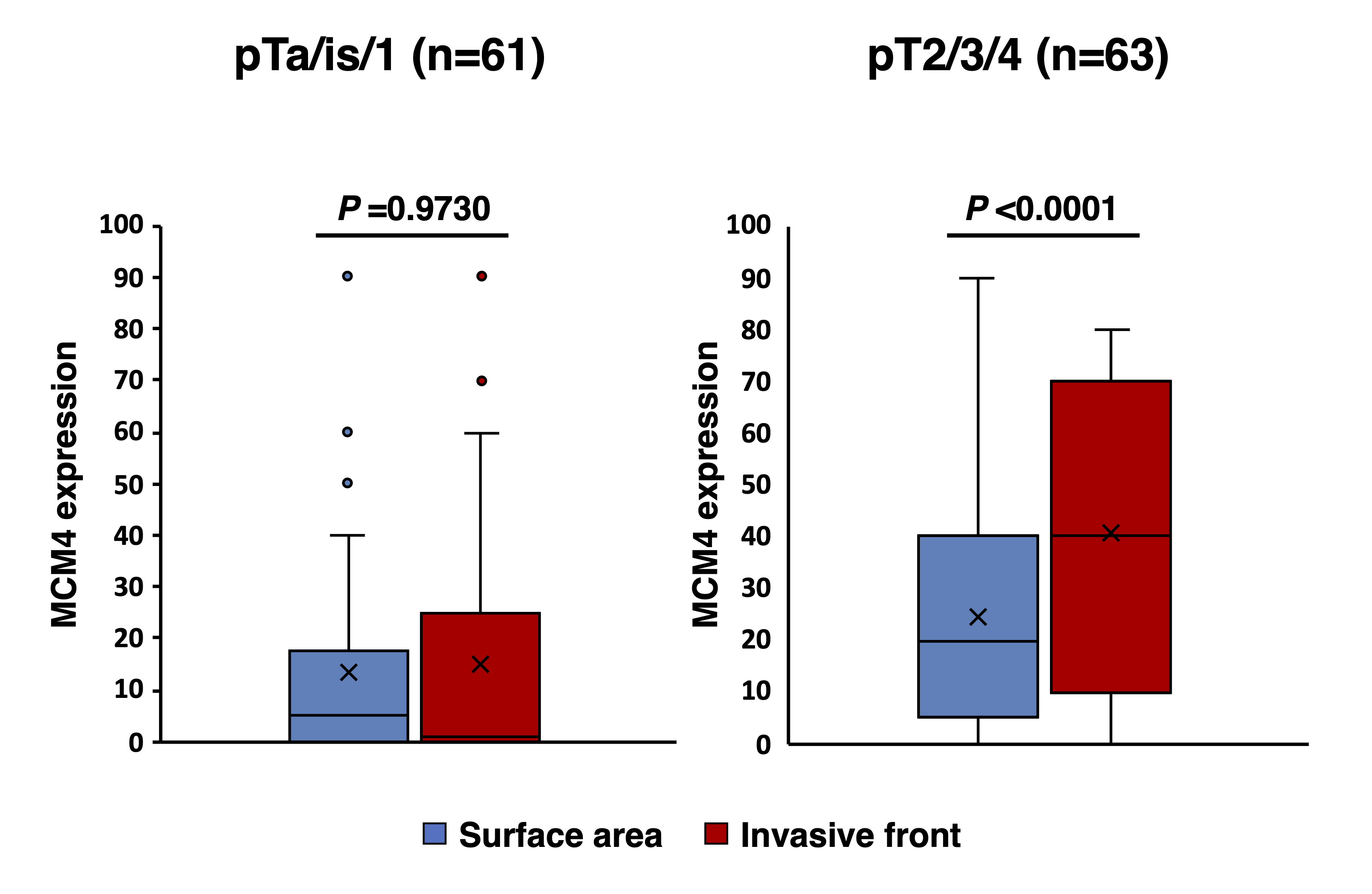

Supplement: Supplementary file 2 — Additional file 2: Supplementary Fig. 1. Comparison of MCM4 expression between the surface area and invasive front in pTa/is/1 (n = 61) and pT2/3/4 (n = 63) tumors respectively. [file 13000_2023_1392_MOESM2_ESM.tiff]

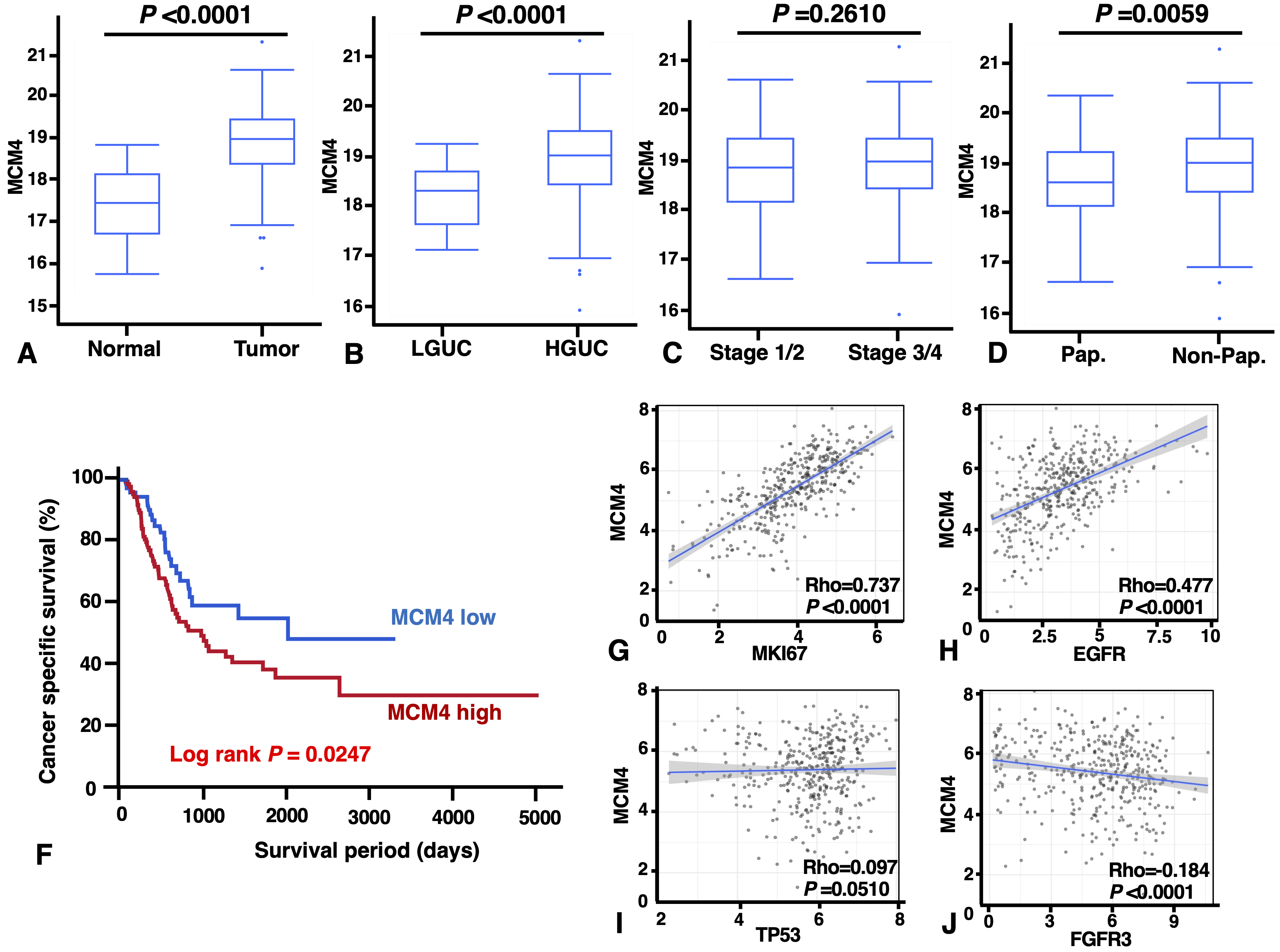

Supplement: Supplementary file 3 — Additional file 3: Supplementary Fig. 2. MCM4 expression in bladder cancer (BC) using TCGA dataset. (A) MCM4 expression in normal and tumor tissues. (B, C, D) MCM4 expression associated with clinicopathological features. Statistical significance was determined by the Mann-Whitney U test. (E) Kaplan-Meier analysis of BC patients with high and low MCM4 expression. (G, H, I, J) In silico analysis of the correlation between MCM4, MKI67, EGFR, TP53 and FGFR3 gene expression. Statistical significance was determined by the Spearman rank correlation test. [file 13000_2023_1392_MOESM3_ESM.tiff]
